# From heat stress to recovery: proteomic insights into endangered *Brachymystax tsinlingensis* survival strategies and the ameliorative effects of anti-stress additives

**DOI:** 10.1007/s44154-025-00270-5

**Published:** 2025-12-31

**Authors:** Zhenlu Wang, Peng Liu, Yizhou Wang, Kaiyong Lan, Zhuo Liu, Xingchen Guo, Huan Ye, Zhipeng Chu, Yu Li, Haibo Jiang, Zhigang Li, Miao An, Jian Shao

**Affiliations:** 1 Laboratory of Fishery Resources and Environmental Protection, College of Animal Science, Key Laboratory of Animal Genetics, Breeding and Reproduction in the Plateau Mountainous Region, Guizhou University, Guiyang, 550025 China; 2 Key Laboratory of Freshwater Biodiversity Conservation, Ministry of Agriculture and Rural Affairs, Yangtze River Fisheries Research Institute, Chinese Academy of Fishery Sciences, Wuhan, 430223 China; 3 Fisheries Research and Technology Extension Center of Shaanxi, Xi’an, 710086 China; 4 Fishery Workstation of Baoji County, Baoji, 721000 China; 5 Special Fisheries Research Institute, Guizhou University, Guiyang, 550025 China

**Keywords:** *Brachymystax tsinlingensis*, Heat stress, Proteomic, Trehalose, Blood–brain barrier

## Abstract

The endangered cold-water fish *Brachymystax tsinlingensis* (*B. tsinlingensis*) serves as a critical sentinel species for aquatic ecosystem responses to climate change. This study investigates heat stress impacts on behavior, physiology, and molecular homeostasis in *B. tsinlingensis*, and evaluates neuroprotective effects of anti-stress additives (vitamin C, gamma-aminobutyric acid, trehalose). Behavioral analysis showed a significant increase in center-zone entries under heat stress. Physiological assays showed a reduction in superoxide dismutase (SOD) and catalase (CAT) activities, alongside an upregulation of *heat shock protein 70* (*Hsp70*), *glucose-regulated protein 78* (*GRP78*), and *caspase* expression under heat stress, with a return to baseline levels following a 12 h recovery. For assays in the brain, histopathological examination identified vacuolation in cerebral tissue after heat stress. Quantitative proteomics analysis identified 831 differentially expressed proteins (DEPs) out of 8,955 proteins during the temperature change process. Pathway analysis revealed that the 'DNA replication' and 'Citrate cycle' pathways were inhibited by heat stress but reactivated during recovery, whereas the 'ECM-receptor interaction' and 'Cell adhesion molecules' pathways exhibited opposite trends. Intervention with additives showed that trehalose enhanced SOD, glutathione peroxidase (GPX), and CAT activities, as well as gene expression related to cell adhesion and barrier function. Gamma-aminobutyric acid maximally suppressed stress-related genes (*Hsp70*, *long-chain-fatty-acid-CoA ligase*, and *arachidonate lipoxygenase*), while vitamin C exhibited general but less targeted effects. As the first proteomic study on *B. tsinlingensis*, this work reveals that blood–brain barrier reconstruction and energy reallocation are key neural survival strategies under temperature change stress. Furthermore, trehalose demonstrates high potential as an anti-heat stress additive by enhancing both antioxidant defenses and blood–brain barrier integrity. These findings advance our understanding of heat adaptation mechanisms in cold-water fish species and provide scientific foundations for conserving *B. tsinlingensis.*

**Supplementary Information:**

The online version contains supplementary material available at 10.1007/s44154-025-00270-5.

## Introduction

Anthropogenic activities have elevated global mean temperature by 0.2°C per decade, with projections indicating a 1.8–4°C rise by 2100 (Forster et al. 2023; IPCC 2022). This irreversible warming, coupled with frequent extreme heat events, poses severe thermal stress to aquatic ectotherms. In aquaculture, heat stress disrupts physiological homeostasis, impairing growth, immunity, and survival in diverse species such as shellfish (Stevens and Gobler 2018), shrimp (Yohana et al. 2024), and teleost fish (Yan et al. 2023). Although systemic responses have been explored, the neural regulatory mechanisms—particularly brain-centric adaptations—remain poorly characterized, especially in cold-water species.

*Brachymystax tsinlingensis* Li, 1966 (*B. tsinlingensis*) is a Quaternary glacial relict and the world's southernmost salmonid. It serves as a critical sentinel for assessing climate impacts on aquatic ecosystems (Xia et al. 2021; Xing et al. 2015). With a restricted thermal niche (optimal: 17 °C), it is highly vulnerable to warming. Listed as endangered in China since 1998 due to habitat fragmentation and overfishing (Sung et al. 1998), its recent advances in captive breeding (Guo et al. 2024a, b, c; Ma et al. 2024) are now threatened by escalating thermal extremes. Our previous study has found that *B. tsinlingensis* has a certain degree of self-healing ability during temperature fluctuations (Wang et al. 2025b). And, our preliminary work in this study revealed behavioral anomalies at 21 °C, suggesting cerebral dysregulation. Critically, to the best of our knowledge, the effects of thermal fluctuation on brain proteome remodeling in this species remain entirely uncharacterized.

Thermal stress profoundly disrupts homeostasis in cold-water fish. Here, we combined behavioral observation, enzymatic assays, gene expression, histopathology, and quantitative proteomics to investigate brain response mechanisms in juveniles of the endangered *B. tsinlingensis*. Furthermore, we evaluate three neuroprotective additives: Vitamin C, a confirmed antioxidant (Islam et al. 2024a; Nikjoo et al. 2023; Rathore et al. 2023); Gamma-aminobutyric acid (GABA), an inhibitory neurotransmitter with anti-stress properties (Islam et al. 2024b); and trehalose, a disaccharide that stabilizes biomolecules under stress (Rihacek et al. 2024). This is the first study to validate their efficacy in mitigating heat-induced neural damage in *B. tsinlingensis*.

## Results

### Effects of temperature change on behavioral and physiological indices in *B. tsinlingensis*

**Fig. 1:**
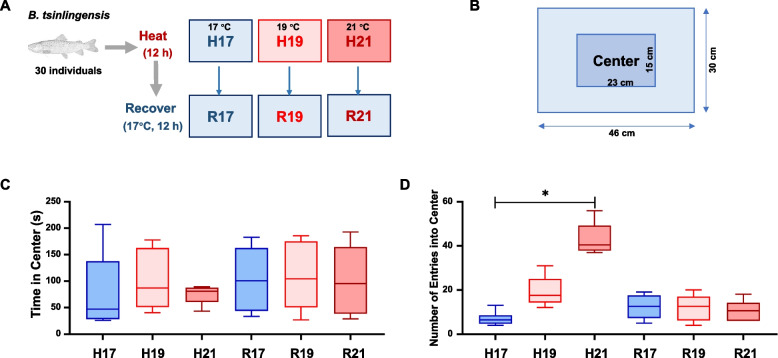
Behavioral responses of *B. tsinlingensis* during temperature changes. **A** Schematic of the experimental design. **B** Top-view diagram of the behavioral testing tank. **C** Time in the center zone. **D** Number of entries into the center zone. Data represent mean ± SD. (*n* = 6). Asterisks (*) denote significant differences (*p* < 0.05, *T* test)

The behavioral responses of *B. tsinlingensis* were evaluated through heat stress and recovery assays (Fig. 1A). A total of 30 fish were distributed into three groups: a control group maintained at 17 °C (H17), a heat stress group maintained at 19 °C (H19), and another heat stress group maintained at 21 °C (H21). Following 12 h of heat stress, the behavioral activities were video-recorded. Mortality was observed exclusively in the group exposed to 21 °C (H21 group).

Behavioral analysis revealed no significant differences in time spent in the center zone across groups (Fig. 1C). However, the number of entries into the center significantly increased with temperature during heat stress (H21 > H17, P < 0.05), then declined in R19 and R21 groups during recovery to levels comparable with R17 (Fig. 1D). These results suggest that exposure to 21 °C significantly altered behavioral patterns of *B. tsinlingensis*.

**Fig. 2:**
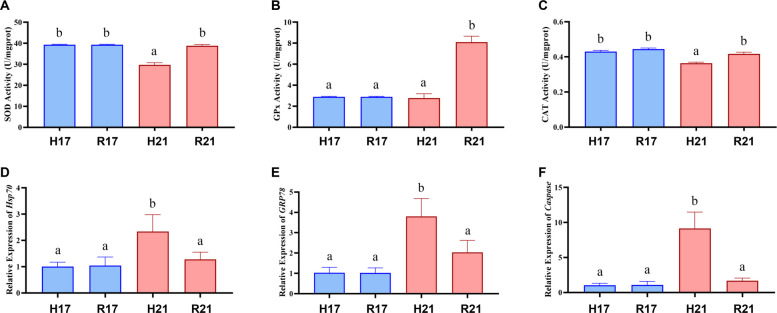
Biochemical response of *B. tsinlingensis* during temperature changes*.* **A** the activity of SOD. **B** the activity of GPX. **C** the activity of CAT. **D** Relative expression of *HSP70*. **E** Relative expression of *GRP78*. **F** Relative expression of *Caspase*. The bars represent the mean ± S.D. (*n* = 3). Different lowercase letters indicate significant differences among groups (*p* < 0.05, one-way ANOVA with Tukey's post-hoc test)

Based on these findings, 21 °C was selected for further stress assessment via stress response index, including SOD, GPx, and CAT activities, as well as the gene expression of heat shock protein 70 (HSP70), glucose-regulated protein 78 (GRP78), and caspase. The absence of significant differences between the H17 and R17 groups confirmed that procedural handling (e.g., tank transfer) did not induce measurable stress. Under heat stress, SOD and CAT activities in the H21 group were significantly lower than those in the H17 group. After a 12 h recovery, these activities returned to levels comparable to those observed in the H17 group. In contrast, the expression levels of *HSP70*, *GRP78*, and *Caspase* were significantly upregulated during heat stress but decreased to control levels after recovery. Additionally, GPx activity remained unchanged from control levels during heat stress but exhibited a significant increase above control levels during recovery (Fig. 2).

### Impact of temperature change on brain of *B. tsinlingensis*

#### Histopathological changes

**Fig. 3:**
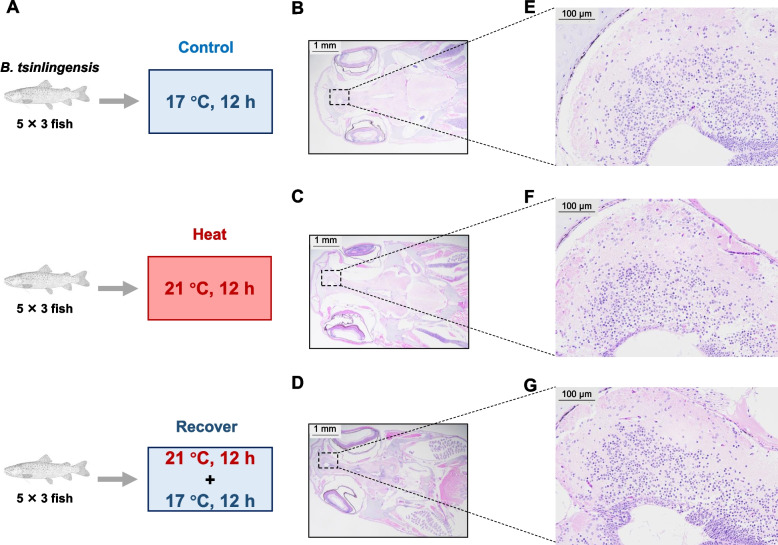
Histopathological responses of *B. tsinlingensis* during temperature changes. **A** Schematic of the experimental design. **B**, **C**, and **D** present brain tissue sections of the control, heat stress, and recovery groups at 20× magnification. **E**, **F**, and **G** present brain tissue sections of the control, heat stress, and recovery groups at 200× magnification

In this study, the histopathological changes in the brain tissue of *B. tsinlingensis* in response to temperature change have been examined. The results showed that the C group exhibited well-organized laminae with densely packed cells (Fig. 3B, E). Mild vacuolation was observed in the H group (Fig. 3F). While the tissue sections from the R group showed reduced vacuolation (Fig. 3G). These findings demonstrate the self-repairing capacity of brain tissue under stress induced by temperature changes.

#### Proteomic profiling

**Fig. 4:**
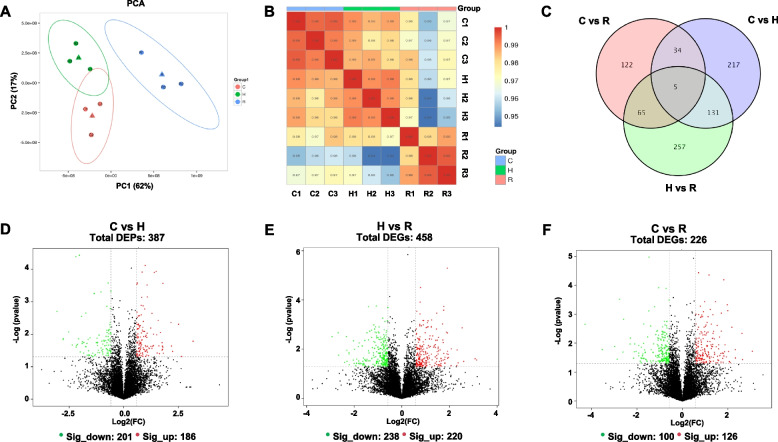
Changes of proteomic in *B. tsinlingensis* during temperature change*.* **A** Principal component analysis. **B** Pearson correlation analysis. **C** Venn analysis. **D**, **E**, and **F** present Volcano plots of DEPs in C vs. H, H vs. R, and C vs. R, respectively

A total of 8,955 proteins were identified from nine brain samples (C1-C3, H1-H3, R1-R3). The results of PCA and correlation heatmap demonstrated high intra-group homogeneity, confirming significant temperature-driven proteome alterations (Fig. 4A-B).

Since GSEA can reveal pathway dysregulation through coordinated gene expression shifts, including non-DEGs with biological relevance, it was applied to all KEGG-annotated proteins. The top 10 significantly enriched pathways for each comparison are detailed in Table 1. In C vs. H (C is control), ‘Ribosome’ (ko03010) was the most significant pathway, with a normalized enrichment score (NES) = −1.655, indicating this pathway was downregulated under heat stress. And ‘Neuroactive ligand-receptor interaction’ (ko04080) was most upregulated (NES = 1.479). During the recovery phase (H vs. R), ‘Salmonella infection’ (ko05132) showed the most significant upregulation (NES = 1.459), and ‘Ribosome’ (ko03010) exhibited marked downregulation (NES = −1.430).

**Table 1:** GSEA-based pathways analysis

Group	ID	Description	setSize	enrichmentScore	NES	*p* value
C vs H	ko03010	Ribosome	127	−0.42612	−1.655	0.001976
	ko04080	Neuroactive ligand-receptor interaction	43	0.45278	1.479	0.036093
	ko00513	Various types of N-glycan biosynthesis	24	−0.55058	−1.560	0.036609
	ko00510	N-Glycan biosynthesis	30	−0.49499	−1.471	0.040777
	ko03030	DNA replication	25	−0.53860	−1.523	0.041096
	ko05168	Herpes simplex virus 1 infection	115	0.32693	1.264	0.079681
	ko01230	Biosynthesis of amino acids	103	−0.33210	−1.255	0.087891
	ko04520	Adherens junction	98	0.34011	1.275	0.088660
	ko03420	Nucleotide excision repair	32	−0.45280	−1.366	0.091255
	ko04216	Ferroptosis	40	0.41422	1.325	0.102941
H vs R	ko05132	Salmonella infection	219	0.33393	1.458682	0.005376
	ko03010	Ribosome	127	−0.36717	−1.43027	0.020513
	ko00790	Folate biosynthesis	19	0.60023	1.644127	0.021231
	ko04370	VEGF signaling pathway	46	0.45928	1.544699	0.022965
	ko04010	MAPK signaling pathway	197	0.30957	1.331541	0.037037
	ko04260	Cardiac muscle contraction	75	0.37035	1.390899	0.040724
	ko04261	Adrenergic signaling in cardiomyocytes	147	0.31964	1.326749	0.045570
	ko04512	ECM-receptor interaction	56	−0.40423	−1.37281	0.056159
	ko00563	Glycosylphosphatidylinositol	13	−0.60246	−1.48523	0.064639
	ko00830	Retinol metabolism	18	−0.53685	−1.42686	0.071154

#### Pathway enrichment analysis of DEPs

In this study, protein expression levels were compared across different groups, resulting in the identification of 387, 458, and 226 DEPs in the comparisons of C vs. H, H vs. R, and R vs. C, respectively (Fig. 4C). The distribution of DEPs was visualized using volcano plots, revealing that the C vs. H contained 201 down-regulated and 186 up-regulated DEPs (Fig. 4D). The H vs. R showed 238 down- and 220 up-regulated DEPs (Fig. 4E), and the R vs. C exhibited 100 down- and 126 up-regulated DEPs (Fig. 4F).

**Fig. 5:**
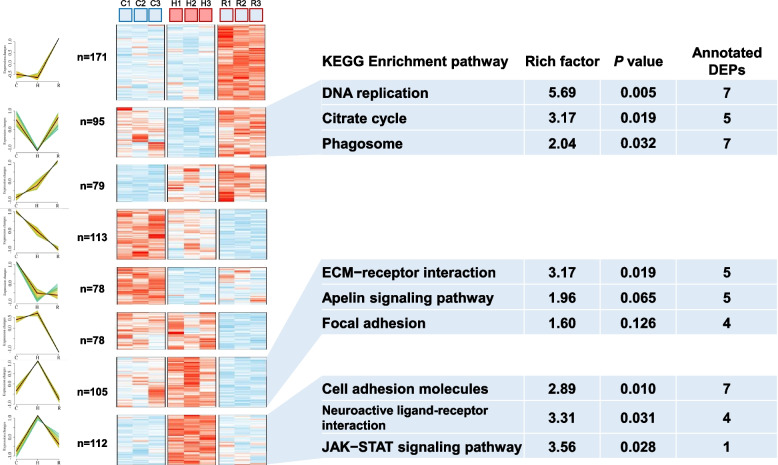
Differentially expression proteins (DEPs) clustering heatmap of the KEGG pathway. Heatmap of 831 DEPs were grouped into eight clusters by K-mean method. The raw value was normalized into Z-score, and the mapping has been colored according to their Z-score

A cluster analysis was conducted on the total of 831 DEPs identified. Within these, the DEPs in cluster 2 (*n* = 95) were found to be down-regulated during heat stress and up-regulated during recovery. In addition, DEPs in cluster 7 and cluster 8 exhibited inverse trends. KEGG pathway enrichment analysis showed that the ‘DNA replication’ was the most significantly enriched pathway in cluster 2, followed by the ‘Citrate cycle’ and ‘Phagosome’ pathways. In cluster 7 and cluster 8, ‘ECM-receptor interaction’ and ‘Cell adhesion molecules’ was the most significantly enriched pathway, respectively (Fig. 5).

### Impact of anti-stress additives on *B. tsinlingensis *under heat stress

Based on the above results of enzyme activity, gene expression, tissue analysis, and proteomics of *B. tsinlingensis* during temperature changes in this study, and combined with previous studies on anti-stress additives, vitamin C (V), GABA (G), and trehalose (T) were selected as potential anti-heat stress additives. Their neuroprotective effects were evaluated in heat-stressed brains.

**Fig. 6:**
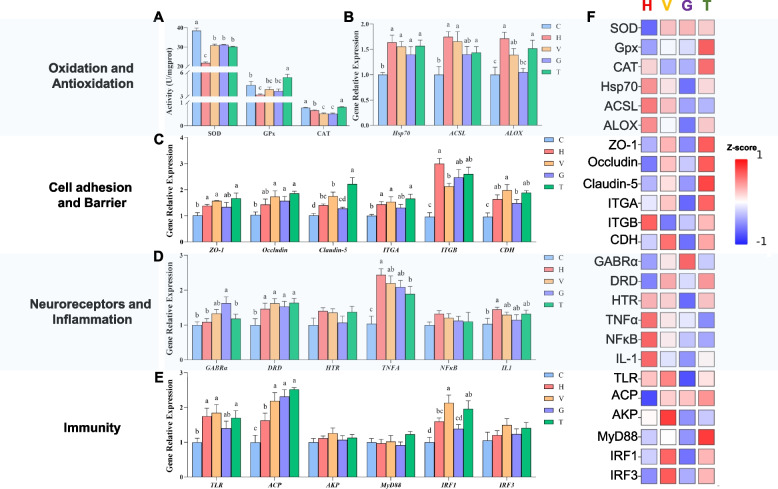
Impact of Anti-stress Additives on *B. tsinlingensis* Under heat stress. **A** The enzyme activities related to antioxidant. **B**, **C**, **D**, and **E** present the relative expressions of genes related to oxidative stress, cell adhesion and barrier function, neural receptors and inflammation, and immunity, respectively. The bars represent the mean ± S.D. (*n* = 3). Different lowercase letters indicate significant differences among groups (*p* < 0.05, one-way ANOVA with Tukey's post-hoc test). **F** The heat map of biochemical parameters under heat stress. The values represent the average fold changes under heat stress (H, V, G and T) versus the control group, with normalized into Z-score, and the mapping has been colored according to their Z-score

The results showed that the antioxidant enzyme activities (SOD, GPx, CAT) were significantly reduced in the H group compared to those in the C group. Compared with the H group, the activities of three enzymes were significantly increased in the T group, while SOD and GPx increased but CAT decreased in the V and G groups (Fig. 6A). Oxidative stress-related genes, including *HSP70*, *long-chain-fatty-acid-CoA ligase* (*ACSL)*, and *arachidonate lipoxygenase* (*ALOX*), were significantly up-regulated in the H group relative to the C group. In each additive group, these expressions showed a declining trend relative to the H group, with the lowest levels observed in the G group (Fig. 6B).

Among genes related to cell adhesion and barrier function, the expressions of *Zonula occluden-1* (*ZO-1*), *Occludin*, *Claudin-5*, *Integrin alpha* (*ITGA*), *Integrin beta* (*ITGB*), and *Cadherin* (*CDH*) in the H, V, G, and T groups were higher than those in the C group, with ZO-1, Occludin, Claudin-5, and ITGA ranked as T > V > H. While the ITGB and CDH expression peaked in H and V groups, respectively (Fig. 6C).

Among the genes related to neural receptors and inflammation, GABRα expression was notably higher in the G group. *Dopamine receptor* (DRD) and *Tumor necrosis factor α* (*TNFα*) expressions were significantly increased in the H, V, G, and T groups relative to the C group. In addition, *Interleukin-1* (*IL-1*) was significantly upregulated in the H group but unchanged in all additive groups when compared with the C group (Fig. 6D).

Among the genes related to immunity, the expression of *Toll-like receptor* (*TLR*) and *Interferon regulatory factor 1* (*IRF1*) in the H, V, and T groups was significantly higher, whereas their expression in the G group did not differ significantly when compared with those in the C group. In addition, the expression of *Acid phosphatase* (*ACP*) in H and all additive groups was significantly higher than those in the C group, and the expression of ACP in all additive groups was significantly higher than those in the H group (Fig. 6E).

## Discussion

**Fig. 7:**
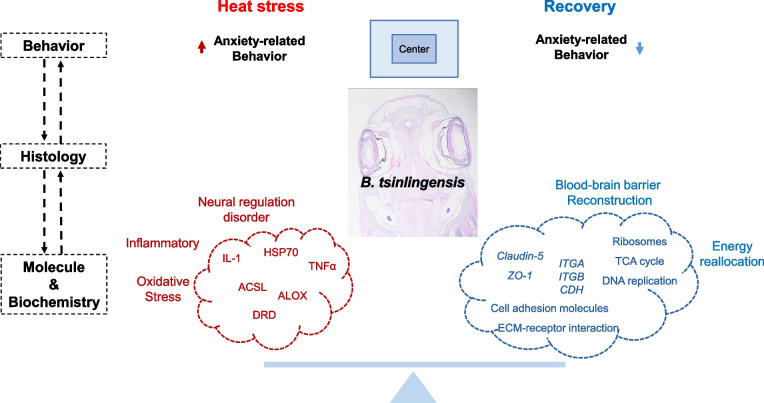
Overview of the homeostatic regulation mechanism of *B. tsinlingensis* during temperature change

*B. tsinlingensis* is an endangered cold-water fish species that is particularly sensitive to temperature changes (Fang et al. 2023). This study first observed abnormal behavior under heat stress, then targeted the brain as the primary tissue of interest to investigate its response mechanisms to temperature changes and the potential protective effects of anti-heat stress additives on brain tissue. These findings contribute to a better understanding of the impacts of heat stress on cold-water fish species and provide scientific basis for the conservation of this endangered species (Fig. 7).

### Heat stress induces brain damage in *B. tsinlingensis*, leading to abnormal neuroregulatory pathways and behavior

Studies indicate that heat stress may cause brain damage in fish through multiple pathways, including oxidative stress, inflammatory responses, and apoptosis (Wang et al. 2023a). In this study, enzyme activity assays of the whole body (Experiment 2) and brain (Experiment 4) both showed that under heat stress, the activity of SOD, GPX, and CAT—which represent the organism’s antioxidant capacity (Jomova et al. 2024)—was significantly downregulated. These results indicate that the antioxidant system of the *B. tsinlingensis* is impaired under heat stress.

Additionally, HSP70 is a common biomarker of stress levels under heat stress (Kaur et al. 2025), and ACSL and ALOX are key regulatory factors in lipid peroxidation and cell death (Klasson et al. 2022; Zheng et al. 2020). In this study, heat stress at 21 °C led to a significant upregulation of HSP70, ACSL, and ALOX expression in the brains of *B. tsinlingensis*. These findings confirmed that thermal stress damages the brain's antioxidant defenses, resulting in oxidative stress injury. Furthermore, TNFα and IL-1 are typical inflammatory factors (Dinarello 2000). The present study found that increased expression of TNFα and IL-1 in brain tissue under heat stress, as well as the vacuolization of brain tissue observed in histological examination results. This inflammatory response, coupled with histological observations, collectively demonstrated damage at both molecular and histological levels.

The brain functions as the central nexus for neural and behavioral regulation, and damage to the brain directly results in abnormalities in neural regulation (Yuan and Bi 2019). Dopamine (DA) transmission has been demonstrated to play a crucial role in the regulation of mood and anxiety-related behaviors (Mata-Bermudez et al. 2024). In this study, we propose a mechanistic link between dopaminergic dysregulation and anxiety-like behavior under heat stress. The significant upregulation of DRD gene expression, coupled with the proteomic upregulation of the ‘Neuroactive ligand-receptor interaction’ pathway, suggests an enhancement of dopaminergic signaling. This hyperactive state is known to induce heightened exploratory or anxiety-like behavior in animals (He et al. 2025; Zhang et al. 2023). Consistent with this molecular profile, behavioral assays revealed a significant increase in the number of entries into the center zone—a validated indicator of elevated anxiety levels (Çevik et al. 2025)—in fish exposed to 21 °C heat stress compared to the control group. Thus, the observed behavioral shift not only represents a clear phenotypic manifestation of neurological dysregulation in *B. tsinlingensis* but is also mechanistically explained by the aberrant activation of the dopaminergic system.

In summary, heat stress induces brain damage in *B. tsinlingensis* through oxidative stress and inflammatory pathways, leading to abnormal neural regulation and ultimately manifesting as anxiety-like behavior.

### BBB reconstruction and energy reallocation are survival strategies in *B. tsinlingensis* during temperature change

The blood–brain barrier (BBB), composed of brain microvascular endothelial cells, is a critical protective mechanism for the central nervous system (Kadry et al. 2020). At the pathway level, the ‘ECM-receptor interaction’ pathway provides basement membrane support, and ‘Cell adhesion molecules’ mediate intercellular anchoring. Together, they contribute to the structural integrity of the BBB, thereby establishing the physical foundation for normal neural receptor activity (Aggarwal et al. 2018; Wang et al. 2025a). In this study, proteomic analysis revealed significant upregulation of both the ‘ECM-receptor interaction’ and ‘Cell adhesion molecules’ pathways under heat stress. This indicates that *B. tsinlingensis* actively enhances basement membrane support and cell–cell anchoring to counteract barrier damage. We propose this prompt response is a key neuroprotective strategy in the brain.

Furthermore, at the molecular level, integrins (ITG) facilitates cell-extracellular matrix interactions (Campbell and Humphries 2011), and cadherins (CDH) mediate cell–cell adhesion (Li et al. 2018), both contributing to BBB formation and maintenance. Additionally, ZO-1, Occludin, and Claudin-5 are major tight junction proteins essential for BBB integrity (Guo et al. 2017). In this study, the gene expression levels of *ZO-1*, *Claudin-5*, *ITGA*, *ITGB*, and *CDH* were found to be significantly increased under heat stress. This phenomenon may be indicative of a compensatory repair mechanism of the BBB, aimed at maintaining barrier integrity during thermal challenge. Moreover, the ‘Apelin signaling pathway’ and the ‘JAK-STAT signaling pathway’, which has been demonstrated to contribute to the maintenance of neuronal homeostasis (Guo et al. 2024b; Wang et al. 2015), were also activated under heat stress. The findings suggest that under conditions of heat stress, the brain activates multi-level neuroprotective mechanisms. This coordinated response confirmed that BBB reconstruction likely represents a key survival strategy employed by *B. tsinlingensis* in resisting heat-induced damage.

On the other hand, the energy reallocation mechanism is also one of the strategies employed by the *B. tsinlingensis* to cope with temperature change. Ribosomes, the molecular machines responsible for protein synthesis within cells, are a critical component of cellular processes (Hurt et al. 2023). DNA replication is a critical process for cell growth and reproduction (Lemmens and Lindqvist 2019), and the ‘Citrate cycle’ (TCA cycle) is a vital metabolic pathway in aerobic respiration, playing a central role in energy production and biosynthesis (Rui 2014). In this study, proteomic analysis via GSEA showed significant downregulation of the Ribosome pathway (ko03010) under heat stress. Further clustering analysis of DEPs revealed inhibition of both the ‘DNA replication’ and ‘Citrate cycle’ pathways under heat stress, followed by upregulation during the recovery phase. Therefore, we inferred that the suppression of these high-energy-consumption metabolic processes represents an active energy optimization strategy by *B. tsinlingensis*. Previous studies have found that shrimp (Wang et al. 2020) and sea cucumbers (Yang et al. 2024) also adopt a strategy of downregulating basal energy metabolism when respond to temperature change stress.

Integrating the aforementioned results, we propose that the *B. tsinlingensis* maintains brain homeostasis under heat stress through a dual adaptive strategy. On the one hand, it downregulates basal metabolism in order to conserve energy, on the other hand, it prioritizes energy allocation to critical processes such as BBB reconstruction.

### Alleviating effects of anti-stress additives on heat stress induced brain damage

The application of anti-stress additives to aquaculture systems is a common practice for alleviating stress-induced damage (Fan et al. 2023; Guo et al. 2025). The potential of vitamin C, GABA, and trehalose as anti stress additives for environmental stress has been reported (Guo et al. 2024a; Nawaz et al. 2022; Ruenkoed et al. 2023), and this study first verified the effectiveness of these three additives in alleviating heat damage in *B. tsinlingensis*.

In this study, the groups that supplementation with vitamin C, GABA, and trehalose all exhibited a significant increase in SOD enzyme activity and ACP gene expression levels in the brains of heat-stressed *B. tsinlingensis*. SOD and ACP are established biomarkers for antioxidant capacity and immune response in fish, respectively (Li et al. 2023; Miao et al. 2025). These findings suggest that these three additives could enhance the antioxidant and immune capabilities of the brain under heat stress to a certain extent. However, a comparative analysis revealed distinct mechanistic differences among them.

Notably, the alleviating mechanisms differed among the three additives. In this study, the genes associated with stress levels (*HSP70*, *ACSL*, and *ALOX*) all exhibited the lowest values in the GABA-added group. Previous research has demonstrated that GABA’s ability to alleviate anxiety-like responses, as well as to mitigate stress reactions in fish under temperature or ammonia stress (Lee et al. 2023; Ogun et al. 2025; Wang et al. 2023b). The GABA function of alleviating neural stress damage observed in this study is consistent with the functions of GABA reported in previous studies.

Our observation pertained to the finding that, the trehalose-added group not only significantly elevated SOD, GPx, and CAT activities, but also maximized expression of cell adhesion and barrier-associated genes (*ZO-1*, *Occludin*, *Claudin-5*, *ITGA*). These findings suggested that trehalose supplementation could enhances antioxidant capacity and promotes the repair of the blood–brain barrier in the brain of *B. tsinlingensis* under heat stress. Although all three additives conferred protection, trehalose demonstrated a unique dual efficacy by simultaneously bolstering antioxidant defenses and reconstructing BBB integrity, distinguishing it from the other additives. While trehalose’s stress-protective effects are well-documented in plants and mammals (Liu et al. 2025; Sevriev et al. 2024), this study provides the first evidence of its dual mechanistic action in fish. In comparison, vitamin C exhibited general but less targeted effects in this study.

These findings establish the theoretical foundation for developing a stress-resistant treatment specifically for the *B. tsinlingensis* and have practical significance for the protection of this endangered species. However, this study is subject to limitations concerning the single-concentration design and organ-specific focus. The optimal dosage, tissue targeting, and underlying mechanisms of each additive require further investigation.

## Conclusion

This study provides the first proteomic profiling of *B. tsinlingensis* under heat stress, revealing key neural adaptation strategies centered on blood–brain barrier restructuring and energy reallocation. In addition, trehalose was identified as a promising neuroprotective additive to support conservation efforts for this endangered species. However, a limitation of this study is its focus on brain tissue and use of a single additive concentration; future dose–response and multi-organ studies would provide further insights.

## Materials and methods

### Experimental fish and culture conditions

The experimental subjects were domesticated, artificially propagated offspring of *B. tsinlingensis* (177 individuals, 5-month-old, average body length: 4.19 ± 0.25 cm), which were randomly selected from a reserve farm in Shaanxi, adhering to our established protocol (Guo et al. 2024c).

### Experimental design and sampling

#### Experiment 1: behavioral observation

The behavioral responses of *B. tsinlingensis* were evaluated though heat stress with subsequent recovery phase. A total of 30 fish were distributed into three groups within 20 L flow-through tanks: a control group maintained at 17 °C (H17), a heat stress group maintained at 19 °C (H19), and another heat stress group maintained at 21 °C (H21). Following 12 h of heat stress, the behavioral activities were video-recorded. For analysis, six randomly selected fish per group were analyzed for the duration and frequency of entries into the central quadrant (central 25% area) during a 5 min observation period (Fig. 1B). During the recovery phase, these fish were relocated to new flow-through tanks (maintained at 17 °C). After an additional 12 h, the same behavioral recording and analysis procedures were conducted.

Throughout the experiment, water quality parameters were maintained with dissolved oxygen > 7 mg/L, pH 7.5, and ammonia (NH₃) < 0.01 mg/L to ensure standardized environmental conditions.

#### Experiment 2: stress response assessment

Forty-two fish were randomly assigned to two groups: a control group maintained at 17 °C and an experimental group maintained at 21°C. Both groups were subjected to the heat stress and recovery protocol previously established in Experiment 1. During the heat stress phase, each group was divided into three replicate tanks (20 L), with seven fish per tank. After 12 h of heat exposure, three fish were randomly selected from each tank, surface-cleaned with phosphate-buffered saline (PBS), and flash-frozen in liquid nitrogen. Among these, one fish was reserved for gene expression analysis, and the other two fish were pooled for enzymatic activity assays.

During the recovery phase, the remaining fish were transferred to new flow-through tanks (maintained at 17 °C). After a 12 h recovery, three fish per tank were collected, flash-frozen, and processed for enzymatic activity and gene expression analyses.

#### Experiment 3: analysis of brain regulatory mechanisms

Building upon the findings of Experiments 1 and 2, which demonstrated that heat stress at 21 °C affects the behavior of *B. tsinlingensis* while also indicating partial self-recovery capacity, this experiment focused on the regulation of the brain's homeostasis under temperature change, given its critical role as the center for behavioral control.

In accordance with protocols in Experiment 2 and our previous study (Wang et al. 2025b), 45 juveniles were distributed into three groups, each with triplicate tanks containing five fish per tank: the heat stress group exposed to 21 °C for 12 h (H group); the recovery group exposed to 21 °C for 12 h followed by a 12 h recovery period at 17 °C in new tanks (R group); and the control group maintained at 17 °C for 12 h (C group) (Fig. 3 A). The water quality parameters were consistent with those in Experiment 1.

At each sampling point, four fish were randomly selected per tank. Among these, one fish was fixed in 4% paraformaldehyde for histological analysis, and the brains of another three fish were sampled, rapidly frozen in liquid nitrogen, and transported on dry ice to Biomarker Technologies (Beijing, China) for proteomic analysis.

#### Experiment 4: anti-stress investigation

This test aimed to evaluate the ameliorative effects of three additives (vitamin C, GABA, and trehalose) on *B. tsinlingensis* subjected to heat stress. All additives were purchased from Yeyuan Biological Technology Co., Ltd. (Shanghai, China). The specific product details are as follows: L-ascorbic acid (vitamin C, CAS# 50–81-7, purity ≥ 99%), gamma aminobutyric acid (GABA, CAS# 56–12-2, purity ≥ 99%), and trehalose (CAS# 99–20-7, purity ≥ 98%). A total of 60 fish were allocated into five treatment groups within 15 tanks (20 L; three tanks per group): a control group maintained at 17 °C (C group), a heat stress group at 21 °C (H group), and three heat stress groups supplemented with vitamin C (V group), GABA (G group), and trehalose (T group), respectively.

The additives were administered at the concentrations recommended for vitamin C, which is a common commercial anti-stress formulations in aquaculture (0.5 mg/L, equivalent to 0.01 g per 20 L tank), with supplementation occurring every 12 h during the 24 h heat exposure. Post-exposure, four fish per tank were collected, followed by flash-frozen in liquid nitrogen after surface-rinsed with PBS. After that, the brains of every two fish were collected and mixed into one sample for subsequent enzymatic activity and gene expression analyses, respectively. Water quality parameters were maintained in accordance with previous test conditions.

### Sample processing and assays

#### Enzyme activity analysis

Samples were homogenized at a ratio of 1:10 (w/v) in ice-cold 0.9% NaCl solution and subsequently centrifuged at 3000 × g for 15 min at 4 °C to collect the supernatant from each sample. The supernatants were then assayed for superoxide dismutase (SOD), glutathione peroxidase (GPX), and catalase (CAT) activities using commercial kits purchased from Nanjing Jiancheng Bioengineering Institute (Nanjing, China), adhering strictly to the manufacturer's protocols, with each sample performed in technical duplicate. Absorbance measurements were obtained using a microplate reader (Thermo Fisher, USA).

#### Gene expression analysis

Total RNA was extracted using the RNAiso Plus reagent (Takara, Japan). First-strand cDNA synthesis was conducted with 1 µg of high-quality total RNA using the PrimeScript™ RT Reagent Kit (Takara). Quantitative real-time PCR (qRT-PCR) analyses were performed on a CFX Connect™ Real-Time System (Bio-Rad, USA) using SYBR® Premix Ex Taq™ II (Takara). The qRT-PCR cycling protocol consisted of: initial denaturation at 95 °C for 30 s; 40 cycles of 95 °C for 5 s, 60 °C for 30 s, and 72 °C for 30 s; followed by melting curve analysis. Each sample was run in technical triplicate. Gene-specific primers for target genes and the reference gene (β-actin) are detailed in Table 2. Relative gene expression was calculated using the 2 ^−ΔΔCt^ method.

**Table 2:** The primers used in this study

Gene Symbol	Gene Name	Forward Primer	Reverse Primer
β-actin	Beta-actin	ACGGACAGGTCATCACCATCGG	TGGAGTTGTAGGTAGTCTCGTGGATTC
HSP70	heat shock 70 kDa protein	CAGTGGGAAGACAGAGAAGAAG	CCCTTCAGGCAGATCAAACT
GRP78	Glucose-regulated protein 78	GATGTTTCCCTCCTGACCATT	GTCCTTGCCTGTCTTCTTCTT
Caspase	Caspase	GACTGACATACACTTTGTCCAACTAC	CTCCTGAGTCCCATAGTTACCATAC
ACSL	Long-chain-fatty-acid-CoA ligase	GGGAGTATCAGTGGATGTCGTAT	GATGATGTGGGTGATCTCTGTTTC
ALOX	Arachidonate lipoxygenase	CGACGGAGATGAAGAAAGGAAAC	ATGAGTGCCCAGTAGATGGTAG
ZO-1	Zonula occluden-1	GTACCCAAAGGCCTGATAGTAATG	ATAAGACAGCACCACAACAGATAAA
Occludin	Occludin	CACCCTGCATCGCTTCAATATC	TCAGATTGCATACTCTCTTCTCTACTC
Claudin-5	Claudin-5	GATGTTTGGTGTCAACGAGAGAG	GGCTCATTCGTATTGTTTAGTGATATTG
ITGA	Integrin alpha	CAACCTCAGCCAGTTTGATACC	GACTCTCCATCCGTCACGATTA
ITGB	Integrin beta	TAGCTGGGATCTACCAACCTAAC	TCCAAGAGGATGGAGGACAATAG
CDH	Cadherin	CAGAGAAGTCAACGCCATACATAAC	CTCACAAAGGTAGGTCTCGTACTC
GABRα	Gamma-aminobutyric acid receptor subunit alpha	TGGGTCTCCTTCTGGATCAATAG	GGTCAGTCTCTGTGTCTTGTTTG
DRD	Dopamine receptor	GACACTGCTGGGAAACCTTATG	CGCTGATAATGCAGAGGTTGAG
HTR	5-Hydroxytryptamine receptor	GTCGGGTGAGAATGACAAAGAAA	CCATTTGTTCAGGACTTGGTAGAG
TNFα	Tumor necrosis factor α	GATTGCAGCAGAGTGATGGTAG	GGCGTGCCAGGTAATGAAA
NFκB	Nuclear factor-kappa-B	CTGTGACAAGGTCCAGAAAGATG	GCTCGTCTCGTTGTCTGATTTAC
IL-1	Interleukin-1	CCAAACTGTCCCACATCAAGAG	TTACTAGAGCAGAGCCAAAGAAATAAG
TLR	Toll-like receptor	GACAAGACCAAACAGTGGGATAAG	TACCACTACGCTGAGAAAGATAGG
ACP	Acid phosphatase	CATGGACAGGACAGGTTCTTTATC	CGTTTATTCAGGAGGGCTTTACC
AKP	Alkaline phosphatase	ACGGACATCACGAGGGTAAA	GTTCCCATATAAGATGGCGGTAAAG
MyD88	Myeloid differentiation primary response 88	CCTCCTTTCAGATGGAGTTCCT	TCTCAGTGATGAGGACGAAAGG
IRF1	Interferon regulatory factor 1	GAACGTCTGCTCTAAGCCAAATAA	TCTGTGCTGTCTACTATGCTCTC
IRF3	Interferon regulatory factor 3	CAAATGCCCGGAACCACTATATC	CACCTCAACCATGACCAGTTTC

#### Histological analysis

After fixation in paraformaldehyde, samples underwent processing through graded ethanol dehydration and paraffin embedding. Subsequently, sections of brain tissue (5 µm) were prepared using a microtome (Leica, Germany) and stained with hematoxylin and eosin (H&E). The stained tissue sections were examined with a Nikon microscope (Tokyo, Japan) to assess histological alterations in the brain of *B. tsinlingensis*.

#### Quantitative proteomics analysis

Samples were analyzed using data-independent acquisition (DIA) proteomic profiling at Biomarker Technologies (Beijing, China). Briefly, the primary procedures included: 1) total protein extraction. 2) peptide preparation. 3) library construction and 4) protein identification and quantification.

Of these, for library construction, high-performance liquid chromatography (HPLC) fractionation was performed using a Rigol L3000 HPLC system. Liquid chromatography-tandem mass spectrometry (LC–MS/MS) analysis was conducted on a Thermo Fisher U3000 UHPLC system coupled with an Orbitrap Fusion mass spectrometer (Thermo Fisher). Data-dependent acquisition (DDA) was applied to the mix-sample for transition library construction, while data-independent acquisition (DIA) was employed for individual samples. For protein identification and quantification, data analysis and visualization for both DDA and DIA were performed using the Proteome Discoverer 2.4 (PD 2.4, Thermo Fisher) platform, Biognosys Spectronaut (version 13), and the R statistical framework. The details of procedures are shown in the Supplementary Information.

### Statistical analysis

For the analysis of enzyme activity and gene expression data, statistical evaluations were conducted utilizing SPSS version 26.0. Prior to conducting group comparisons, the assumptions of normality and homogeneity of variances were assessed and confirmed. Comparisons between two experimental groups were executed using an independent samples t-test, whereas comparisons involving three or more groups were analyzed using ANOVA, followed by Tukey's post hoc test. Statistical significance was determined at a threshold of *p* < 0.05 for all tests. Data are reported as mean ± standard deviation (S.D.). Figures were generated using GraphPad Prism version 9.

For proteomics data, analysis was performed on the BMKCloud platform (www.biocloud.net), which included protein functional annotation, differential protein expression analysis, and pathway analysis. Protein functions and pathways were annotated according to the NCBI Non-Redundant Protein Sequences (NR) databases and the Kyoto Encyclopedia of Genes and Genomes (KEGG) databases. Differentially expression proteins (DEPs) were identified based on fold change (FC) thresholds (FC > 1.5 for up-regulation or FC < 1/1.5 for down-regulation with *p* < 0.05). Additionally, analyses such as principal component analysis (PCA), heatmaps, Venn diagrams, volcano plots, clustering analysis, and gene set enrichment analysis (GSEA) were performed using the R-3.1.1 software packages available on the Biocloud online platform.

## Supplementary Information

Supplementary Material 1.

## Data Availability

The datasets during and/or analysed during the current study available from the corresponding author on reasonable request.

## Abbreviations

B. tsinlingensis: Brachymystax tsinlingensis
GABA: Gamma-aminobutyric acid
SOD: Superoxide dismutase
GPX: Glutathione peroxidase
CAT: Catalase
β-actin: Beta-actin
HSP70: Heat shock 70 kDa protein
GRP78: Glucose-regulated protein 78
Caspase: Caspase
ACSL: Long-chain-fatty-acid-CoA ligase
ALOX: Arachidonate lipoxygenase
ZO-1: Zonula occluden-1
Occludin: Occludin
Claudin-5: Claudin-5
ITGA: Integrin alpha
ITGB: Integrin beta
CDH: Cadherin
GABRα: Gamma-aminobutyric acid receptor subunit alpha
DRD: Dopamine receptor
HTR: 5-Hydroxytryptamine receptor
TNFα: Tumor necrosis factor α
NFκB: Nuclear factor-kappa-B
IL-1: Interleukin-1
TLR: Toll-like receptor
ACP: Acid phosphatase
AKP: Alkaline phosphatase
MyD88: Myeloid differentiation primary response 88
IRF1: Interferon regulatory factor 1
IRF3: Interferon regulatory factor 3
