# Supplementary material for: From heat stress to recovery: proteomic insights into endangered Brachymystax tsinlingensis survival strategies and the ameliorative effects of anti-stress additives
Source: Stress Biol. 2025 Dec 31;5(1):77. doi: 10.1007/s44154-025-00270-5 (PMC12753618; doi:10.1007/s44154-025-00270-5)
Supplement: Supplementary file 1 — Supplementary Material 1. [file 44154_2025_270_MOESM1_ESM.docx]

**From Heat Stress to Recovery: Proteomic Insights into Endangered** ***Brachymystax tsinlingensis* Survival Strategies and the Ameliorative Effects of Anti-Stress Additives.**

Zhenlu Wang^1,5#^, Peng Liu^1,5#^, Yizhou Wang^1^, Kaiyong Lan^1^, Zhuo Liu^1^, Xingchen Guo^1^, Huan Ye^2^, Zhipeng Chu^2^, Yu Li^3^, Haibo Jiang^1^, Zhigang Li^4^, Miao An^1,5^, Jian Shao^1^^,5*^

^1^Laboratory of Fishery Resources and Environmental Protection, College of Animal Science, Key Laboratory of Animal Genetics, Breeding and Reproduction in the Plateau Mountainous Region, Guizhou University, Guiyang, 550025, China;

^2^ Key Laboratory of Freshwater Biodiversity Conservation, Ministry of Agriculture and Rural Affairs, Yangtze River Fisheries Research Institute, Chinese Academy of Fishery Sciences, Wuhan 430223, China;

^3^Fisheries Research and Technology Extension Center of Shaanxi, Xi’an 710086, China

^4^Fishery Workstation of Baoji County, Baoji, 721000, China;

^5^Special Fisheries Research Institute, Guizhou University, Guiyang 550025, China

*Correspondence:

shaojian5098@163.com (J. Shao)

^#^These authors contributed equally to this work.

**Quantitative proteomics analysis**

**1 Total Protein Extraction**

Samples were minced individually with liquid nitrogen and lysed in lysis buffer containing 50mM NH_4_HCO_3_ pH 7.4, 10 mM MgCl_2_, 7 M urea, 2 M thiourea, followed by 5 min of ultrasonication on ice. The lysate was centrifuged at 12000 g for 15 min at 4°C and the supernatant was transferred to a clean tube. Protein concentration was determined by Bradford protein assay.

**2 Peptide Preparation**

100μg protein from each sample were reduced with 10mM DTT for 1 h at 56°C, and subsequently alkylated with sufficient Iodoacetamide for 1 h at room temperature in the dark. The protein was digested with Trypsin Gold (Promega) at 1:50 enzyme-to-substrate ratio. After 16 h of digestion at 37°C, part of peptides from samples were mixed equally. The mixture sample (mix-sample) and the remaining peptides (single-sample) were all desalted with C18 cartridge to remove the high urea, and desalted samples were dried by vacuum centrifugation.

**3 Library Construction**

**3.1 HPLC Fractionation**

The mix-sample was fractionated using a C18 column (Waters BEH C18 4.6×250 mm, 5 μm) on a Rigol L3000 HPLC operating at 1 mL/min, the column oven was set as 50°C. Mobile phases A (2% acetonitrile, adjusted pH to 10.0 using ammonium hydroxide) and B (98% acetonitrile, adjusted pH to 10.0 using ammonium hydroxide) were used to develop a gradient elution. The solvent gradient was set as follows: 3%B, 5min; 3-8% B, 0.1 min; 8-18% B, 11.9 min; 18-32% B, 11 min; 32-45% B, 7 min; 45-80% B, 3 min; 80% B, 5 min; 80-5%, 0.1 min, 5% B, 6.9 min. The eluates were monitored at UV 214 nm, collected for a tube per minute and merged into 6 fractions finally. All fractions were dried under vacuum and reconstituted in 0.1% (v/v) formic acid (FA) in water. Add 0.2 μL standard peptides to the fraction sample for subsequent analyses.

**3.2 LC-MS/MS Analysis-DDA mode**

For transition library construction, shotgun proteomics analyses were performed using an U3000 UHPLC system (Thermo Fisher) coupled with an Orbitrap fusion mass spectrometer (Thermo Fisher) operating in the data-dependent acquisition (DDA) mode. A sample volume containing 1 μg of total peptides from the fraction sample reconstituted in 0.1% FA was injected onto a home-made C18 Nano-Trap column (2 cm×100 μm, 3 μm). Peptides were separated on analytical column (25 cm×75 μm, 100 A), using a 120 min linear gradient from 0 to 100% of eluent B (0.08%FA in 80% ACN,20% water) in eluent A (0.1%FA in water) at a flow rate of 350 nL/min. The detailed solvent gradient listed as follows: 0-4% B, 8 min; 4-10% B, 11 min; 10–25% B, 88 min; 25–50% B, 98min; 50–99% B, 102 min; 99–0% B.

The Orbitrap Fusion mass spectrometer was operated in the data-dependent acquisition mode using Xcalibur3.0 software and there is a single full-scan mass spectrum in the Orbitrap (250-1450 m/z, 120,000 resolution) followed by 3 seconds data-dependent MS/MS scans in an Ion Routing Multipole at 30% normalized collision energy (HCD).

**3.2 LC-MS/MS Analysis-DIA mode**

The single-sample was reconstituted in 0.1% FA, mixed with 0.2 μL standard peptides (iRT kit, Biognosys), and injected onto U3000 UHPLC system (Thermo Fisher) coupled with an Orbitrap fusion mass spectrometer (Thermo Fisher) operating in the data-independent acquisition (DIA) mode. The liquid conditions were the same as above. For DIA acquisition, MS1 resolution was set to 120000, and MS2 resolution was set to 30000. The m/z range covered from 350 to 1350 m/z and variable 60 cycles. Full scan AGC target was set to 4×106, injection time to 50 ms. DIA settings were NCE 35%, target value 1×106 and maximum injection time was set to auto to allow the mass spectectometer always operating in the parallel ion filling and detection mode.

**4 The identification and quantitation of protein**

Data analysis and visualization of DDA and DIA data were performed using Proteome Discoverer 2.4 (PD 2.4, thermo) platform, Biognosys Spectronaut version 13, and R statistical framework. DDA MS raw files were analyzed by PD software (version 2.4) and peak lists were searched against protein database. Cysteine carbamidomethylation was set as a fixed modification and N-terminal acetylation and methionine oxidation as variable modifications. The false discovery rate was set to 5% for proteins and peptides, respectively and was determined by searching a reverse database. The enzyme specificity was set to trypsin (enabling cleavage before proline), and a maximum of two missed cleavages were allowed in the database search. Peptide identification was performed with an allowed initial precursor mass deviation up to 10 ppm and an allowed fragment mass deviation of 10 ppm. MS1-based label free quantification (LFQ) was done using maxLFQ algorithm.

MS2-based-label free quantification was carried out by analyzing DIA raw data using Biognosys Spectronaut (version 13) software. Briefly, data extraction and extraction window were set to “dynamic” with correction factor 1, identification was set to “normal distribution p-value estimator” with q-value cutoff of 0.01. The profiling strategy was set to “iRT profiling” with qvalue cutoff of 0.01. Ultimately, protein inference was set to “from search engine”, protein quantity was set to “Average precursor quantity” and smallest quantitative unit was set to “Precursor ion” (summed fragment ions).
